# Editorial: Understanding how myeloid cell development and function meet tissue distinct metabolic requirements

**DOI:** 10.3389/fimmu.2024.1373468

**Published:** 2024-02-07

**Authors:** Erika M. Palmieri, Robert J. Salmond, Alessio Menga

**Affiliations:** ^1^ Cancer Innovation Laboratory, National Cancer Institute (NCI), Frederick, MD, United States; ^2^ Leeds Institute of Medical Research at St James’s, University of Leeds, Leeds, United Kingdom; ^3^ Department of Molecular Biotechnology and Health Sciences, Molecular Biotechnology Center “Guido Tarone”, University of Torino, Torino, Italy

**Keywords:** macrophage, immunometabolism, microenvironment, tissue niche, myeloid cell, cross talk, metabolic pathway

Myeloid cells play pivotal roles in regulating tissue equilibrium and immune responses, undergoing significant metabolic changes as they transition between different states. The emerging field of immunometabolism underscores how alterations in cellular metabolism influence immune cell behavior by regulating genetic processes (1). This Research Topic delves into comprehending how myeloid cell development and function align with the unique metabolic demands of various tissues.

The generation of inflammatory signaling molecules, activities aimed at eliminating microbes, and the discharge of reactive oxygen species (ROS) are connected to the process known as “classical” activation (M1) in macrophages. In contrast, “alternative” activation (M2), plays a role in facilitating the healing process, triggering humoral responses, and contributing to the progression of tumors. These two primary activation states demonstrate distinct metabolic preferences, relying on glycolysis and oxidative phosphorylation, respectively. While the importance of metabolism in myeloid cell function is recognized, the *in vivo* relevance of these observations remains unclear. Studies highlight complex metabolic footprints in macrophages activated under various stimuli and tissue contexts (2).

In an effort to close this knowledge gap, in our Research Topic Gorshkova et al. delve into the immune characteristics of naked mole rats (NMRs) compared to mice, focusing on macrophages. Activated NMR macrophages displayed a pro-inflammatory phenotype but limited nitric oxide (NO) production compared to mouse macrophages. The study highlighted differences in metabolic responses, suggesting that NMRs might have evolved unique metabolic adaptations that influence their immune functions, oxidative stress responses, and NO production during inflammation. Unlike typical M2 (anti-inflammatory) responses observed in mouse macrophages, NMR macrophages poorly differentiated under standard conditions, suggesting species-specific adaptations influencing their immune phenotype. The study highlighted peculiarities in NMR genomes related to immune response genes, such as alterations in arginine metabolism and absence of certain genes associated with anti-inflammatory responses in mice. Overall, while the production of NO by murine macrophages has already been “uncoupled” from a proinflammatory phenotype (3), the study provides insights into how the immune system of NMRs differs from mice, shedding light on potential evolutionary adaptations and mechanisms behind NMR’s unique immune characteristics, which could influence its longevity and disease resistance.

As nicely reviewed by Chen and Tang, macrophages act as “homeostatic controllers,” responding to environmental signals to regulate tissue functions. They discuss how within a tissue, parenchymal cells like hepatocytes, cardiomyocytes, and splenic fibroblasts, perform essential functions specific to each organ while non parenchymal cells, including macrophages, contribute to tissue repair, substance metabolism, and immune responses. The interaction between these cell types orchestrates organ functions and ensures systemic physiological balance. The authors also elaborate on intestinal macrophages and their role in maintaining mucosal integrity and regulating gut motility as well as on the collaboration between alveolar macrophages (AM) and alveolar epithelial cells (AEs) as vital for lung function. AM are key immune defenders that continuously capture and metabolize inhaled pathogens and particles, preventing excessive inflammation. They also regulate surfactant levels to maintain lung biomechanics while AEs influence AM phenotypes through cytokines and exosomes, modulating lung homeostasis. However, in chronic obstructive pulmonary disease (COPD), the accumulated oxidized lipids in AEs may impair AMs’ phagocytic ability, leading to inflammation and lung damage.

On this subtopic, work by Verma et al. reported in our Research Topic, details an example of hypercytokinemia and lethal lung damage in acute respiratory distress syndrome (ARDS), highlighting a substantial role of mononuclear phagocytes. In a model of influenza A virus and methicillin-resistant Staphylococcus aureus coinfection, utilizing conditional knockout mouse, they highlight that IFNγ signaling in myeloid cells alone is enough to trigger ARDS pathogenesis. The study emphasizes the significance of necrotic cell death due to phagocyte oxidative burst in causing acute lung injury and that dysregulated inflammatory responses, rather than increased cell infiltration, are crucial in IFNγ-driven lung damage.

The impact of immune dysfunction is particularly important also in clinical situations like radiation exposure, where severe infections affect radiation injury prognosis. Macrophages, being more resistant to radiation compared to neutrophils, play a significant role in innate immune responses after radiation injury. The work from Yamaga et al. uncovers the role of triggering receptor expressed on myeloid cells 1 (TREM-1) in myeloid cells as fundamental for tissue health and prognosis. Authors reveal that ionizing radiation increases TREM-1 levels in mouse macrophages, with eCIRP (a cellular stress response protein) released from irradiated macrophages being a potential cause. TREM-1 may exacerbate organ injury and might be linked to pyroptosis and the eCIRP-TREM-1 axis might impair macrophage function, affecting bacterial phagocytosis, a critical aspect during infections post-radiation.

Despite their fundamental role in immune function, abnormalities in myeloid cell development or function can result in cancerous conditions within the body. De Luca et al. explore circulating cell-free DNA (cfDNA) as a potential prognostic marker in Myelofibrosis (MF), a rare blood cancer characterized by clonal myeloproliferation. Authors found a direct relationship between higher cfDNA levels and indicators of advanced disease stages, adverse genetic mutations, unfavorable cytogenetic abnormalities, and poorer survival rates. The inability of the bone marrow to produce healthy blood cells might lead to cell death and subsequent release of DNA into circulation. Various mechanisms of cell death appear to be involved, suggesting that systemic DNA leakage from the malfunctioning myeloproliferative neoplasm (MPN) clone or inflammasome-mediated processes might contribute to elevated cfDNA levels. The role of monocytes is emphasized since these might be a significant source of IL-18 production in MF, indicating their contribution to the inflammatory milieu of the disease. The study implies that inhibiting inflammasome-related processes in monocytes could be a potential therapeutic strategy to mitigate disease progression.

When looking at myeloid cells in the context of leukemia, much work has been recently initiated to try to understand and improve prognosis prediction and treatment selection, specifically based on metabolic signatures of cancers. A key metabolic change commonly observed in cancer cells is continuous aerobic glycolysis, leading to the utilization of substantial glucose quantities, even when oxygen is available. Yang et al. in our Research Topic classified patients into high or low-risk groups using a Carbohydrate-Related Genes (CRG) signature consisting of 10 genes, accurately assessing patient survival. This signature was validated across various databases and confirmed as an independent predictor for risk stratification in acute myeloid leukemia (AML) patients. Authors highlight the critical role of carbohydrate metabolism in oncogenesis, emphasizing its significance in providing energy for cell proliferation. They also connect carbohydrate metabolism with cancer progression, focusing on pathways like PI3K/AKT/mTOR, KRAS, MYC, and p53, identifying potential drug targets in carbohydrate metabolism for AML therapy. Additionally, differential gene expression analysis revealed immune response regulation discrepancies between high and low-risk groups, linking carbohydrate metabolism to the immune status in AML. Furthermore, the study discusses about potential implications for immunotherapy response based on the CRG signature, highlighting a suppressed immune microenvironment in high-risk patients.

The impact of the microenvironment, cell interactions, and crosstalk on disease progression is epitomized by the liver milieu. Macrophages, including Kupffer cells (KCs), maintain liver homeostasis but can aggravate conditions like non-alcoholic fatty liver disease (NAFLD) when disturbed. As reviewed by Alabdulaali et al. in the Research Topic, macrophages show diversity and respond differently in NAFLD stages. Their communication with other cells influences inflammation, fibrosis, and disease progression. Liver macrophages’ heterogeneity, originating from different sources and exhibiting diverse functions, including pro-inflammatory (M1-like) and anti-inflammatory (M2-like), is crucial in NAFLD pathogenesis. These macrophage subtypes demonstrate unique metabolic activities and may offer potential therapeutic targets in managing NAFLD. The cooperation between KCs and hepatocytes (HCs) in keeping the liver homeostasis primarily involves KCs overseeing metabolic activities, especially lipid metabolism. This dynamic partnership is also well-explored in this Research Topic by Chen and Tang. KCs play a role in shaping the expression of hepatokines and dampening the ketogenesis pathway in HCs when the body experiences changes in energy levels, such as during fasting or feeding. Additionally, these macrophages can produce exosomes containing insulin-sensitizing miR-690, impacting lipid metabolism and insulin resistance in HCs.

KCs and monocyte-derived macrophages contribute significantly to NAFLD progression. Targeting these liver macrophages emerges as a promising therapeutic avenue, especially in the early stages, potentially reducing damage and progression to more severe conditions like non-alcoholic steatohepatitis (NASH).

Exploring mechanisms that regulate macrophage functions at the transcriptional and epigenetic levels shows promise. Certain compounds like carotenoids and inhibitors of galectin-3 or specific receptors (CCR2/CCR5) in macrophages have shown positive preclinical outcomes in reversing or alleviating NASH-related symptoms.

The role of macrophages in NAFLD involves influencing hepatic lipid accumulation and triggering inflammation. Macrophage lipid processing and signaling indeed play active roles in NAFLD progression. This led research to also focus on molecules secreted from macrophages that have potential paracrine effects. Strategies to inhibit specific molecules like TNF or that target particular macrophage subsets, such as CD206hi ESAM+ KC2, offer potential therapeutic interventions. Most importantly, the interaction between KCs and HCs involves not only cytokines but also cellular metabolites. Recent work indeed has demonstrated a role for macrophage-derived itaconic acid, a product of the immune-responsive gene 1 (IRG1) protein, in acting in trans upon hepatocytes to modulate the liver’s ability to metabolize fatty acids (4).

Taken together, the work in the present Research Topic contributes to unraveling the intricate relationship between myeloid cell metabolism and tissue-specific environments, potentially revolutionizing disease treatment approaches tailored to tissue-dependent immune responses.

## Author contributions

EP: Writing – original draft, Writing – review & editing. RS: Supervision, Writing – review & editing. AM: Supervision, Writing – review & editing.
